# Proteolytic regulation of synaptic plasticity in the mouse primary visual cortex: analysis of matrix metalloproteinase 9 deficient mice

**DOI:** 10.3389/fncel.2015.00369

**Published:** 2015-09-22

**Authors:** Emily A. Kelly, Amanda S. Russo, Cory D. Jackson, Cassandra E. Lamantia, Ania K. Majewska

**Affiliations:** Center for Visual Science, School of Medicine and Dentistry, Department of Neurobiology and Anatomy, University of RochesterRochester, NY, USA

**Keywords:** dendrite, spine, plasticity, ocular dominance, primary sensory cortex (S1), primary visual cortex (V1), matrix metalloproteinase 9 (MMP9)

## Abstract

The extracellular matrix (ECM) is known to play important roles in regulating neuronal recovery from injury. The ECM can also impact physiological synaptic plasticity, although this process is less well understood. To understand the impact of the ECM on synaptic function and remodeling *in vivo*, we examined ECM composition and proteolysis in a well-established model of experience-dependent plasticity in the visual cortex. We describe a rapid change in ECM protein composition during Ocular Dominance Plasticity (ODP) in adolescent mice, and a loss of ECM remodeling in mice that lack the extracellular protease, matrix metalloproteinase-9 (MMP9). Loss of MMP9 also attenuated functional ODP following monocular deprivation (MD) and reduced excitatory synapse density and spine density in sensory cortex. While we observed no change in the morphology of existing dendritic spines, spine dynamics were altered, and MMP9 knock-out (KO) mice showed increased turnover of dendritic spines over a period of 2 days. We also analyzed the effects of MMP9 loss on microglia, as these cells are involved in extracellular remodeling and have been recently shown to be important for synaptic plasticity. MMP9 KO mice exhibited very limited changes in microglial morphology. Ultrastructural analysis, however, showed that the extracellular space surrounding microglia was increased, with concomitant increases in microglial inclusions, suggesting possible changes in microglial function in the absence of MMP9. Taken together, our results show that MMP9 contributes to ECM degradation, synaptic dynamics and sensory-evoked plasticity in the mouse visual cortex.

## Introduction

Distinct areas of the brain show increased cortical plasticity during defined critical periods of development (Hensch, 2005a, b; Morishita and Hensch, 2008; Erzurumlu, 2010; Maffei et al., 2010; Levelt and Hübener, 2012; Hubener and Bonhoeffer, 2014). This localized increased plasticity requires the coordination of multiple signaling events, including synaptic scaling (Desai et al., 2002), synaptic stabilization and circuit reorganization (Hensch, 2005b). While many mechanisms contribute to the opening and closing of critical periods of plasticity, the restructuring of the extracellular matrix (ECM) may be an important regulator of plasticity. As development proceeds, cells in the brain become gradually encased in an accumulation of structural proteins that form a non-permissive environment for reorganization. The ECM is composed of a lattice of structural proteins, including laminin, tenascins, thrombospondin, and lectins that form a mesh-like network around maturing cells called a perineuronal net (PNN). While the PNN generates stability and can promote cell maturity, it also generates a barrier which can prevent further interactions between neurons and advancing axons, act as a scaffold for the binding of molecules which may then inhibit synaptic formation, and restrict receptor mobility at the synapses, influencing receptor exchange. Thus ECM maturation can limit activity-dependent plasticity at the end of the critical period and into adulthood (Berardi et al., 2004).

The ECM is differentially regulated during development due to the secretion of proteolytic molecules that promote plasticity (Ethell and Ethell, 2007). Matrix metalloproteinases (MMPs) have recently emerged as key players involved in long term memory and the underlying synaptic changes. These proteins constitute a large family of zinc-dependent endopeptidases which can cleave and remodel the ECM and are involved in many physiological and pathological processes (McCawley and Matrisian, 2001; Sternlicht and Werb, 2001). Of the 25 known MMPs, MMP2 and MMP9 are the most prevalent in the brain (Yong, 2005), where they can regulate substrates with roles in synaptogenesis, synaptic plasticity and long-term potentiation—including other proteases, growth factors, cell adhesion molecules, cytokines and neurotransmitter receptors (reviewed in Ethell and Ethell, 2007). MMPs are locally synthesized, secreted from both neurons and microglia, and their activity is regulated by other proteases such as tissue plasminogen activator protein (tPA) and plasmin as well as tissue inhibitors of metalloproteinases (TIMPs). Both tPA and MMP9 have been implicated in the induction of plasticity (Mataga et al., 2002, 2004; Szklarczyk et al., 2002; Nagy et al., 2006, 2007), and may have critical roles at the level of the dendritic spine (Mataga et al., 2004; Oray et al., 2004; Tian et al., 2007; Conant et al., 2010) where local proteolytic activity triggers dendritic remodeling.

The rodent visual system serves as an ideal model to study mechanisms of synaptic plasticity and circuit remodeling (Hensch, 2005a; Tropea et al., 2009). Within the binocular visual cortex, cells exhibit *ocular dominance (OD)*, whereby they respond preferentially to input coming from one eye over the other. Following monocular deprivation (MD), responsiveness shifts from the closed eye to the open eye, and this is called *Ocular Dominance Plasticity* (ODP; Gordon and Stryker, 1996). There are two stages of ODP: first a weakening of deprived eye inputs and reorganization of intracortical connections in the superficial layers (Trachtenberg et al., 2000; Trachtenberg and Stryker, 2001), followed by strengthening of non-deprived eye inputs and anatomical reorganization of thalamocortical afferents (Shatz and Stryker, 1978; Antonini and Stryker, 1993; Antonini et al., 1999; Frenkel and Bear, 2004). While there is evidence that local upregulation of proteases (Mataga et al., 2002, 2004; Oray et al., 2004) and their downstream effects on spine motility and turnover (Mataga et al., 2004; Oray et al., 2004) play an important role in ODP, it is unknown whether protease-mediated ECM remodeling accompanies ODP.

Here, we investigated changes in the ECM during ODP and the contribution of MMP9 to this process. We show that specific components of the ECM are rapidly remodeled and that MMP9 regulates both functional plasticity and ECM remodeling. We also show that loss of MMP9 alters the development of cortical excitatory synapses and induces modifications in microglia. Our results provide evidence for the importance of MMPs in proteolytic regulation of synaptic plasticity *in vivo*.

## Materials and Methods

### Animals

Animals were treated in strict accordance with the University of Rochester Committee on Animal Resources and the 2011 NIH Guide for the care and use of laboratory animals. Mice were group housed with food and water available ad libitum under a fixed 12 h light/dark cycle. The following mouse lines were used in this study: C57Bl/6 (Charles River, Wilmington, MA), green fluorescent protein (GFP)-M (Feng et al., 2000), MMP9 knock-out (KO) (The Jackson Laboratory; B6.FVB(Cg).Mmp9tm1Tvu/J—C57Bl/6 background), and MMP9 KO/GFP-M (generated by crossing GFP-M and MMP9 KO mice, in house).

### Extracellular Matrix Immunohistochemistry

For brain harvesting, mice were anesthetized with sodium pentobarbital (150 mg/kg; i.p.) at P30-P35 and perfused through the aortic arch with ice-cold phosphate-buffered saline (0.1 M PBS, 0.9% NaCl in 50 mM phosphate buffer [pH 7.4]) followed by 4% paraformaldehyde (PFA; in 0.1 M PBS, pH 7.4). Brains were post-fixed in 4% PFA for 2 h and transferred to an increasing gradient of sucrose (10, 20, 30% in ultra-pure water) at 4°C. Brains were sectioned coronally at a 50 μm thickness on a freezing, sliding microtome (Microm; Global Medical Instrumentation, Ramsey, MN).

Fixed brain sections containing visual cortex were immersed in 0.1% sodium borohydride (in 0.1 M PBS) for 30 min at room temperature (RT), washed in 0.1 M PBS, and processed freely floating. For *hyaluronic acid* (HA) detection, sections (ND = 5, 2dMD = 5, 4dMD = 5, 7dMD = 5) were blocked in a solution containing 2% bovine serum albuminin (BSA) and 0.1 M phosphate buffered saline (0.9% NaCl in 50 mM phosphate buffer [pH 7.4]) for 1 h. Sections were then incubated for 72 h in a solution containing biotinylated hyularonic acid binding protein (HABP, 1:200, Seikagaku Biobusiness Corp, Amsbio, UK) in 2% BSA in 0.1 M PBA at 4°C in a humidified chamber. Specific activity was detected using an ABC reagent (1:100; Vector Laboratories Inc, Burlington CA) and visualized with 3, 3-diaminobenzidine (0.5 mg/ml) and hydrogen peroxide (0.03%) in buffer solution (DAB peroxidase kit; Vector Laboratories). For *heparan sulfate proteoglycan* (HSPG) detection, sections (ND = 5, 2dMD = 5, 4dMD = 5, 7dMD = 5) were first processed with a 2% hydrogen peroxide/ 70% methanol antigen retrieval step. Sections were digested with 5 mU/ml of heparitinase (from flavobacterium heparinum, Seikagaku Corporation, Tokyo, Japan, Cat100703) diluted in a buffer containing 100 mM sodium chloride and 1 mM calcium chloride for 3 h at 37°C. Sections were blocked in a solution containing 2% BSA in 0.1 M PBS followed by a primary incubation in anti-∆-heparan sulfate monoclonal antibody (3G10) (1:100, Seikagaku Corp) in a humidified chamber overnight at 4°C. Sections were placed in a secondary incubation containing anti-mouse biotinylated IgG (1:200, Vector Laboratories), 2% BSA in 0.1 M PBS. Specific activity was detected using an ABC reagent (1:100; Vector Laboratories Inc, Burlington CA) and visualized with 3, 3-diaminobenzidine (0.5 mg/ml) and hydrogen peroxide (0.03%) in buffer solution (DAB peroxidase kit; Vector Laboratories). To determine chondroitin sulfate proteoglycan (CSPG) composition, sections were processed for *wisteria floribunda agglutinin* (WFA) which recognizes N-acetylgalactosamine, a sugar that is found in the glycosaminoglycan chains of CSPGs (Murakami et al., 1999). Sections (C57Bl/6: ND = 5, 2dMD = 4, 4dMD = 4, 7dMD = 4; MMP9 KO ND = 5, 2dMD = 5, 4dMD = 5, 7dMD = 5) were processed free-floating by first blocking sequentially in a streptavidin and biotin solution (Biotin/Streptavidin Block Kit, Vector Labs, SP-2002, per kit instructions). Sections were further blocked in a solution containing 3% BSA, 20 mM Lysine, and 0.2% Triton-X in 0.1 M PBS. Sections were incubated in a serum solution containing 1% BSA in 0.1 M PBS and biotinylated-WFA (1:200; Vector Laboratories, B1355) at 4°C in a humidified chamber for 24–48 h. Specific activity was detected using an ABC reagent (1:100; Vector Laboratories Inc, Burlington CA) and visualized with 3, 3-diaminobenzidine (0.5 mg/ml) and hydrogen peroxide (0.03%) in buffer solution (DAB peroxidase kit; Vector Laboratories). Processed tissue sections were mounted out of a solution containing 1% Gelatin/99% ethanol in 0.1 M PB onto clean slides. Once dried, slides were dehydrated in an ascending concentration of ethanol into xylene. Slides were coverslipped using DPX mounting media Electron Microscopy Sciences (EMS).

Brightfield microscopy images were taken on a BX51 Olympus scope at X 10 magnification (UPlanFL N; X 10/0.30;Olympus, Tokyo, Japan) and 40X magnification (UPlanFL N; X 40/0.50; Olympus) mounted with a Spot Pursuit RT color digital camera (Diagnostic Instruments, Sterling Heights, MI, USA). Following image acquisition, images were analyzed using Image J software.^1^

#### Laminar Determination

**Figure 1:**
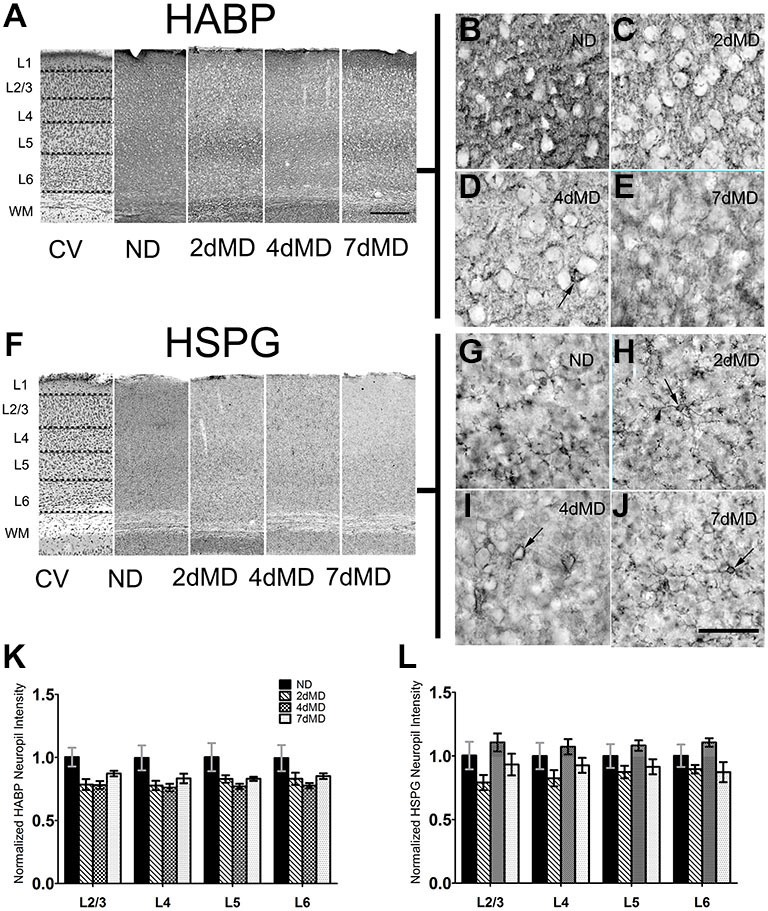
**Hyaluronic acid (HA) and heparin sulfate proteoglycans (HSPGs) are differentially regulated in rodent sensory cortex. (A)** Representative examples of low-mag (4X) images showing laminar demarcation in cresyl violet (CV) alongside HABP immuno-reactive tissue. **(B–E)** hyularonic acid binding protein (HABP) immunohistochemistry in all conditions shows immunoreactivity in neuropil and occasional dense cellular labeling (arrow). **(F)** Representative examples of low-mag (4X) images showing laminar demarcation in CV alongside HSPG immuno-reactive tissue. **(G–J)** Anti-HSPG immunoreactivity displays dense staining around cell bodies (arrows) and in neuropil in all conditions. **(K,L)** HABP labeling intensity in cortical neuropil was altered by deprivation (Two-way ANOVA; main effect of deprivation, *p* < 0.05; Bonferroni *post hoc* analysis, *p* > 0.05 for all comparisons). **(J)** HSPG labeling intensity was unchanged when compared to ND controls suggesting differential regulation of HSPGs following monocular deprivation (MD). Values were normalized to their respective ND controls for presentation purposes only. All values reported are the mean ± standard error of the mean (SEM). Scale bars **(A,F)** = 250 μm; **(B–E,G–J)** = 200 μm.

**Figure 2:**
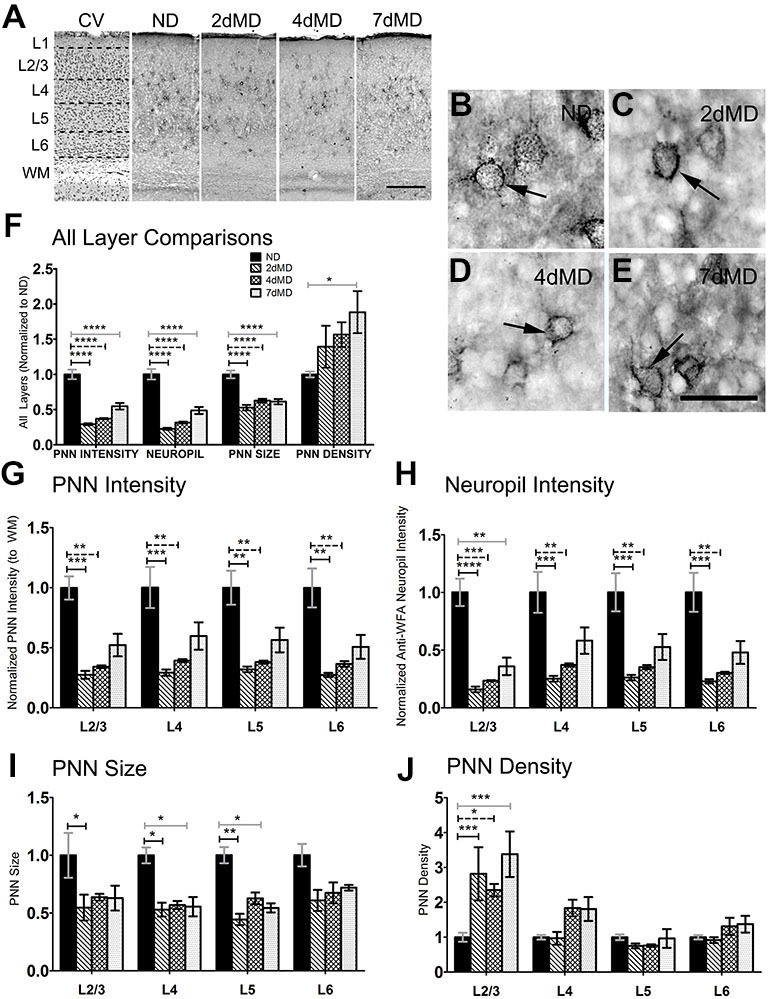
**Chondroitin sulfate proteoglycans (CSPGs) are degraded following MD. (A)** Representative examples of low-mag (4X) images showing laminar demarcation in CV alongside CSPG (anti-wisteria floribunda agglutinin (WFA)) immuno-reactive tissue. **(B–E)** Anti-WFA immunoreactivity labels CSPG deposits in perineuronal nets (PNNs) (arrows) in all experimental conditions (arrows). **(F)** Quantitative analysis of several labeling parameters across all layers in experimental conditions. Values were normalized to ND controls for presentation purposes only. Significant differences following MD in PNN intensity, Neuropil intensity and PNN size were observed. Changes in PNN density were restricted to 7dMD. **(G–J)** Quantitative analysis of individual labeling parameters across layer. Degradation of CSPG immunoreactivity was most prominent in overall PNN intensity **(G)** and surrounding neuropil **(F)** following 2dMD and 4dMD, where deposition parameters often returned to baseline by 7dMD. PNN size **(I)** was significant reduced in L2/3-5 following 2dMD. This size reduction was also evident in L4 and L5 after 7dMD. A laminar specific increase in PNN density **(J)** was noted only in L2/3. Scale bar **(A)** = 250 μm. **(B–E)** = 50 μm. Statistics = **(F)** = One-way ANOVA within each measurement parameter with Bonferroni multiple comparisons *post hoc* analysis; **(G–J)** = Two-way ANOVA with Bonferroni multiple *post hoc* comparisons. All values reported are the mean ± SEM. *p < 0.05, **p < 0.001, ***p < 0.0001, ****p < 0.00001.

For laminar determination, alternating sections were counterstained with a 0.5% Cresyl violet (CV) Acetate stain (1% CV in dH_2_O in an acetate buffer [9:1 ratio of acidic component (0.6% Glacial acetic acid in dH_2_O) to basic component (1.36% sodium acetate in dH_2_O)]. ECM analysis was performed in individual layers (2/3, 4, and 6). Determination of layer was performed based on cellular size and density by an experienced observer using neighboring sections stained with CV. The distinct cytological architecture allowed high magnified image collection at the vertical center of each layer. Layer 2/3 (the external pyramidal layer) contains predominantly small and medium sized pyramidal neurons. Layer 4 (the internal granular layer) contains different types of smaller stellate and pyramidal neurons, providing an obvious cytological transition from L2/3 and a definitive border with L5. L5 (the internal pyramidal layer), contains large pyramidal neurons while L6 (the polymorphic or multiform layer) contains few large pyramidal neurons and many small spindle-like pyramidal and multiform neurons. (Mountcastle, 1997) For examples see Figures 1A,F, 2A, 4A.

### Extracellular Matrix Analysis

All image collection and analysis was done blind to genotype and manipulation. Samples were blinded before imaging and uncoded following the completion of analysis.

#### HA and HSPG Analysis

Images were imported into Image J, background subtracted and overall image intensity measurements (8-bit, 0–255) were taken from the primary binocular visual cortex contralateral to the deprived eye. Background was determined in each image based on the average of multiple areas inside selected cell bodies that appeared unstained or very lightly stained. For each section (five sections per animal), values were normalized to the background subtracted values obtained in the white matter (WM) below layer 6 where staining was present, albeit less intense than in the cortex, and where immunoreactivity was expected to be insensitive to MD to control for staining variance across animals. The values were further normalized to the average ND value for display purposes only.

#### CSPG Analysis

Images were imported into Image J and background subtraction was performed on each image (*n* = 5 per animal). PNNs were identified by an experienced observer. All PNNs were included irrespective of staining intensity as long as they were determined to be in focus in the image. PNNs were manually traced using the tracing tool in ImageJ. Staining intensity of the PNN was collected as well as PNN size. These structures were then removed from the image, a layer mask applied and the neuropil intensity was determined. To control for variability in staining PNN and neuropil intensity values were normalized to the intensity observed in the WM as for HABP and HSPG analysis. PNN density was determined as the number of PNNs observed divided by the area analyzed. For all parameters values were normalized to the average ND value in each condition for display purposes only.

### Intrinsic Signal Optical Imaging

To induce ODP, mice (C57Bl/6: ND = 10, 4dMD = 9, 7dMD = 9; MMP9 KO: ND = 5, 4dMD = 6, 7dMD = 7) were monocularly deprived for 4 and 7 days at the height of the critical period for cortical plasticity. On P28 ± 2, lid margins were resected and lids sutured under isoflurane anesthesia (2–3%). After 4 and 7 days of MD, animals were anesthetized with isoflurane (2–3%) along with chlorprothixene (2 mg/kg) and the sutures were removed for imaging. The skull over visual cortex was cleared, covered with agarose (1%) and a coverslip and illuminated with 700 nm light. Anesthetic level was maintained with isoflurane (0.75%) during imaging. IOS was performed using a DALSA 2 M30 CCD camera (Kalatsky and Stryker, 2003). An image of the vascular pattern was obtained through the skull by illumination with a green filter (550 nm). Intrinsic signal images were then captured using a red filter (700 nm). Visual stimuli consisting of white horizontal square-wave bars on a neutral background moving downward (270°) and upward (90°) for 6 min per run, were presented to each eye separately. The amplitude of the fast fourier transform component in the binocular visual cortex was analyzed offline using Matlab to determine OD (Kalatsky and Stryker, 2003; Tropea et al., 2010). OD was compared between MMP9 deficient mice and C57Bl/6 controls. An ocular dominance index (ODI) was calculated as (contralateral-ipsilateral)/(contralateral + ipsilateral) based on the average pixel intensities of the images obtained during visual stimulation of each eye (Cang et al., 2005). Positive ODI values indicate a contralateral bias; negative values indicate an ipsilateral bias.

### Dendritic Spine Analysis

To examine the effects of MMP9 depletion on dendritic spine density and morphology, MMP9 KO mice were crossed with GFP-M mice (Feng et al., 2000). MMP9KO/GFP (*n* = 6; 657 spines total) mice were compared to GFP-M controls (*n* = 6; 927 spines total). Animals were perfused between P32 and P35. Brains were sectioned on a freezing sliding microtome to a 50 μm thickness. Sections were mounted out of a 0.1 M PBS solution and coverslipped with Prolong Gold (Invitrogen) anti-fade media. Confocal microscopy image acquisition and spine analysis was performed as described previously (Bogart et al., 2011). Briefly, layers 2/3 within the primary somatosensory cortex (S1) were identified for imaging on a Zeiss LSM 510 confocal microscope (Care Zeiss, Thornwood NY). The distributions of imaged areas within S1 were similar between experimental conditions. GFP-labeled brain sections were excited at 488 nm and imaged through an HFT 514/633 dichroic and 530–600 nm band pass filter. Excitation power and settings for pinhole and detector gain were optimized to minimize photobleaching and utilize the full dynamic range of fluorophore emission intensity. High resolution (512 × 512 pixels) confocal image stacks of layer 5 apical dendritic branches located in layer 2/3 were collected using a 100 × oil-immersion lens (NA 1.46), at a digital zoom factor 2 (pixel size 0.082 μm), and a *z*-step of 0.5 μm. Additional *z*-stacks were collected using lower power objectives to document the position of acquired images within the dendritic arbor stacks. Dendritic segments of the primary apical dendrite in layer 2/3 were located between 70 and 150 μm from the pial surface and were selected based on the quality of GFP expression and resulting signal-to-noise ratio, so that spines could be identified and measured as accurately as possible.

Following image acquisition, *z*-stacks were exported to TIF format using Zeiss’s Axiovision software (release 4.6). Image analysis was then done using Image J. To quantify spine density, spines were identified by manually stepping through the *z*-stack, and marked on the projected image. Only spines located in the plane of their parent dendrite branch were marked and counted. Spines falling out of plane and those projecting from the parent dendritic branch in the *z*-dimension were systematically excluded from our counts even if they were visually identifiable as spines. For the purposes of this study we define spines as all visible dendritic protrusions and filopodia are included in the analysis. After all spines on a segment were marked, segment length was measured using the segmented line tool. 3D segment length was accounted for by measuring the absolute difference in depth between the two ends of the segment and using the Pythagorean Theorem. Spine density was then computed as the number of spines per micron of dendrite. Since spine density varies with dendritic diameter (Irwin et al., 2002), we ensured that analyzed dendrites were well matched between the two genotypes. Dendritic diameters were not significantly different between the two groups (CTL ND = 1.628 μm ± 0.148, MMP9 KO = 2.040 μm ± 0.190, Student’s *t*-test, *p* = 0.118). We also analyzed the dimensions of dendritic spines. Spine length was measured on maximum intensity projections using a segmented line tool to draw a line from the most distal point of the spine head to the base of the spine neck where it connects to the parent dendritic branch. Measurements of spine head and neck width were made based on fluorescence measurements. The fluorescence profile of a line placed along the center of the head and neck was determined and fit to Gaussian using custom-written algorithms in MATLAB (The MathWorks, Inc., Natick, MA). The full-width half-max was taken as a measure of spine head width. This method may overestimate the size of small spines that fall under the limit of the resolution of our confocal microscope. The amplitude of the Gaussian fit to the spine neck fluorescence profile was normalized to the amplitude of the fit to the spine head profile as a relative measure of spine neck width. Background fluorescence was subtracted before fitting on a dendrite-by-dendrite basis. Great care was taken to avoid saturation in images, and saturated points were removed from the fluorescence profiles. Spines with more than two saturation points were removed from the analysis as it was determined that accurate fits were obtained if fewer than three points were omitted. This affected less than 2% of the population of spines.

### Two-photon Imaging and Dendritic Spine Turnover Analysis

For two-photon imaging, mice (GFP-M = 5, 1, 432 spines total; MMP9 KO ND = 8, 927 spines total) were anesthetized with a fentanyl cocktail (fentanyl; 0.05 mg/kg of body weight; midazolam; 5 mg/kg; medetomidine; 0.5 mg/kg; i.p.); the skull was exposed, cleaned and glued to a thin metal plate. S1 was identified according to stereological coordinates. The skull above the imaged area was thinned with a dental drill. During surgery and imaging, the animal’s temperature was kept constant at 37°C with a heating pad and anesthesia was maintained with periodic administration of fentanyl. Imaging and data analysis were carried out as previously described (Majewska et al., 2006). A custom-made two-photon scanning microscope (Majewska et al., 2000) was employed, using a wavelength of 920 nm and a 20 × 0.95 NA objective lens (Olympus, Melville, NY) at 8.5× digital zoom. A map of the blood vessels was taken as a reference point. After image acquisition the animal’s scalp was sutured and the animal was allowed to recover before being placed back in its home cage. Four days later, the animal was re-anesthetized and the skull re-exposed. The blood vessels map and dendritic architecture were used to identify the same imaging regions. Dendritic protrusions were identified as persistent if they were located within 0.5 μm laterally on the subsequent imaging session. Elimination and formation rates refer to the numbers of lost spines and new spines, respectively, observed on the second imaging time point divided by the total number of spines present in the first imaging session.

### Immunoperoxidase Reactivity for Electron Microscopy

Sections (C57Bl/6 ND = 3, MMP9 KO ND = 3) were immersed in 0.1% borohydride (in 0.1 M PBS) for 30 min at RT, washed in 0.1 M PBS, and processed freely floating following a pre-embedding immunoperoxidase protocol previously described (Riad et al., 2000; Tremblay et al., 2010b). Briefly, sections were rinsed in 0.1 M PBS, followed by a 2 h pre-incubation at RT in a blocking solution containing 5% normal goat serum and 0.5% fish gelatin. Sections were then incubated for 48 h at RT in rabbit anti-Iba-1 (1:1,000 in blocking solution; Wako Pure Chemical Industries) and rinsed thoroughly in 0.1 M PBS (pH 7.4). Sections were incubated for 2 h at RT in goat anti-rabbit IgG conjugated to biotin (Jackson Immunoresearch) and with streptavidin-horseradish peroxidase (Jackson Immunoresearch) for 1 h at RT in blocking solution. Immunoreactivity was visualized with diaminobenzidine (0.5 mg/ml) and hydrogen peroxide (0.03%) in buffer solution (DAB Peroxidase Substrate Kit; Vector Laboratories). Sections were then post-fixed flat in 1% osmium tetroxide and dehydrated in ascending concentrations of ethanol. They were treated with propylene oxide, impregnated in Durcupan EMS overnight at RT, mounted between ACLAR embedding films EMS, and cured at 55°C for 48 h. Areas of the primary visual cortex (V1, Layer 2), at the level approximating the transverse planes A + 0.16 to A + 0.72 (Franklin and Paxinos, 2008), were excised in a trapezoid shape from the embedding films in a selected orientation to accurately determine the pial surface during ultrathin sectioning and re-embedded at the tip of resin blocks. Ultrathin sections (60–80 nm; evidenced by the sections silver sheen) were cut with an ultramicrotome (Reichert Ultracut E) and collected on bare square-mesh grids.

### Electron Microscopy Imaging and Data Analysis

Eighty pictures were randomly taken at 40,000× in layer 2 of V1 (approximately 10 microns from the pial surface) in each animal at the tissue-resin border corresponding to a total surface of ~1,000 μm^2^ of neuropil per animal (as in Tremblay et al., 2007, 2009, 2010a; Bouvier et al., 2008, 2010; Kelly et al., 2010; Mortillo et al., 2012, among others). Images were captured on a Hitachi 7650 Transmission Electron Microscope using a Gatan 11 megapixel Erlangshen digital camera and Digitalmicrograph software. TIFF images were exported into Adobe Photoshop (CS5.5) and adjusted for brightness and contrast in preparation for analysis. Cellular profiles were identified using a series of criteria previously defined in single-ultrathin sections (Peters et al., 1991; Tremblay et al., 2009, 2010a; Lu et al., 2011). Asymmetrical synapse density and the length of the postsynaptic density (PSD) were analyzed using Image J software (500 μm^2^/animal/genotype; *n* = 3 CTL ND, *n* = 3 MMP9 KO ND). Only asymmetrical synapses that displayed visible neurotransmitter vesicles in the presynaptic terminal and an abutting postsynaptic membrane containing an electron dense PSD were included in the study. Using the line tool in Image J software, a straight line was drawn from edge to edge of the PSD to determine PSD length. Neighboring structures to Iba-1 immunoreactive elements were classified into the following categories: *dendrite (shaft)*, *dendritic spines, putative filopodia*, *axon terminal*, and glial elements (including *microglia* and *astrocytes)*. We conservatively classified all the subcellular profiles that were difficult to identify as “unknown”. See Kelly et al. (2014) for electron microscopy element classification details. To control for the fact that large processes have the ability to interact with more microglial processes and contain more inclusions, we normalized our observations to the size of the microglia processes analyzed.

### Microglial Morphology Analysis

To examine the effects of MMP9 depletion on microglial morphology, C57Bl/6 (CTL, *n* = 6 mice) and MMP9 KO (*n* = 6 mice) brain slices were processed with anti-Iba-1, a microglial marker. For brain harvesting, mice were anesthetized with sodium pentobarbital (150 mg/kg; i.p.) at P32 and perfused through the aortic arch with ice-cold phosphate-buffered saline (0.1 M PBS, 0.9% NaCl in 50 mM phosphate buffer [pH 7.4]) followed by 4% paraformaldehyde (PFA; in 0.1 M PBS, pH 7.4). Brains were post-fixed in 4% PFA for 2 h and transferred to an increasing gradient of sucrose (10, 20, 30% in ultra-pure water) at 4°C. Brains were sectioned coronally at a 50 μm thickness on a freezing, sliding microtome (Microm; Global Medical Instrumentation, Ramsey, MN). Fixed brain sections containing visual cortex were immersed in 0.1% sodium borohydride (in 0.1 M PBS) for 30 min at RT, washed in 0.1 M PBS, and processed freely floating. Sections were blocked in a solution containing 0.5% BSA, 5% normal serum and 0.3% Triton-x for 2 h. Sections were then incubated for 24–48 h in rabbit anti- Iba-1 [ionized calcium binding adaptor molecule-1; microglia- marker; 1:2500; Wako; (Imai et al., 1996)]. Sections were washed in 0.1 M PBS and incubated for 2 h in a solution containing anti-rabbit Alexa 594 (Invitrogen). Sections were mounted out of a 0.1 M PBS solution and coverslipped with Prolong Gold (Invitrogen) antifade media. Z-stack images from layers 2/3 within binocular visual cortex were collected on a Zeiss LSM 510 confocal microscope (Care Zeiss, Thornwood NY, USA) and images were imported into Image J for analysis. The process area, soma area, process/soma length (LA, longest axis of the process/soma area) and process/soma width (SA, perpendicular to the process length) were measured using Image J. The circularity index was based on the following equation: 1−(LA−SA/LA+SA).

#### Statistical Analysis

Statistical analysis was performed using Prism VI statistical analysis software (Graphpad Software, Inc; La Jolla, CA). All values reported are the mean ± standard error of the mean (SEM). For all analyses, significance was based on *α* = 0.05. When comparing between groups, significance was determined using two-tailed unpaired Student *t*-tests and Bonferroni multiple comparison *post hoc* analysis. Initial multi-group comparisons were performed using one-way and two-way ANOVAs. On two occasions, an outlying number was omitted following the use of the Grubbs Outlier Test.^2^

## Results

Degradation of the ECM promotes neurite outgrowth, axon regeneration and functional recovery (Bradbury et al., 2002; Pizzorusso et al., 2002), and may therefore play important roles during experience-dependent plasticity. To determine if ECM composition contributes to plastic changes in the rodent visual cortex, we profiled the degree of degradation of several components of the ECM following MD in the binocular visual cortex. MD was performed within the visual critical period beginning on postnatal day (P)28 (Gordon and Stryker, 1996, for animals numbers throughout the results please See “Materials and Methods” Section). We decided to focus on three important components of the brain ECM: HA, which comprises the fundamental base of the ECM lattice (Yamaguchi, 2000), heparin sulfate proteoglycans (HSPGs) and CSPGs which are strongly associated with PNNs (Deepa et al., 2006). We hypothesized that MD would stimulate ECM degradation to facilitate plasticity. As plasticity is elicited differentially across cortical layers following MD (Trachtenberg and Stryker, 2001; Oray et al., 2004), we performed quantitative analysis of the overall immunoreactivity across cortex and in specific layers to determine possible layer-specific effects of ECM degradation over time following deprivation (Figures 1A,F). Immunohistochemical analysis of HA (using HA binding protein; HABP) displayed a prominent deposition in the neuropil (Figures 1B–E) with occasional densities around cell bodies, similar to previous reports (as shown in Figure 1D; Costa et al., 2007). While levels of HABP immunoreactivity were decreased in all layers at all time points following MD (two-way ANOVA, effect of deprivation *p* < 0.0001), no statistically significant effects were observed in individual layers (Bonferroni *post hoc* tests; *p* > 0.05). This suggests that although deprivation elicits degradation of HA, the effect is subtle. (Figure 1K).

Similarly, HSPG immunoreactivity revealed both pronounced deposition around cell bodies, forming concentrated rings around several cells that extended into proximal neuronal processes (Figures 1G–J), and in the neuropil, resulting in diffuse patterning throughout all layers in all conditions. HSPG deposition was not significantly affected following MD (Figure 1L) suggesting differential regulation of HSPG as compared with HA during MD.

WFA immunoreactivity, indicative of CSPG distribution, was prevalent in PNN structures that thoroughly encased a subset of neuronal cell bodies (Figures 2A–E). This dense perineuronal accumulation was found in all deprivation conditions, where it extended into the surrounding neuropil. Given the fundamental role of CSPG composition in plasticity regulation (Bandtlow and Zimmermann, 2000), and also the more complex nature of its immunoreactivity, we investigated several CSPG immunoreactivity parameters, including PNN staining intensity, PNN size, PNN density (number of PNNs/unit area) and overall neuropil immunoreactivity. Beginning as early as 2dMD, we noted a significant reduction in PNN intensity, PNN size, and neuropil composition (*p* < 0.05–0.001, two-way ANOVA, Bonferroni multiple comparisons *post hoc* analysis) when compared to ND controls (Figure 2F). When analyzed independently, all cortical layers showed significant changes in PNN intensity and neuropil composition at both 2dMD and 4dMD (Figures 2G,H; two-way ANOVA, Bonferroni multiple comparisons *post hoc* analysis) with a recovery by 7dMD. Interestingly, a reduction in PNN size was found primarily after 2dMD in superficial layers which recovered by 7dMD in most layers (Figure 2I). Finally, changes to PNN density were restricted to superficial layers in all MD conditions (Figure 2J). These findings suggest that ECM degradation occurs rapidly following MD during the time when deprived eye inputs weaken, and recovers when plasticity is maximal at 7 days of deprivation (Frenkel and Bear, 2004).

**Figure 3:**
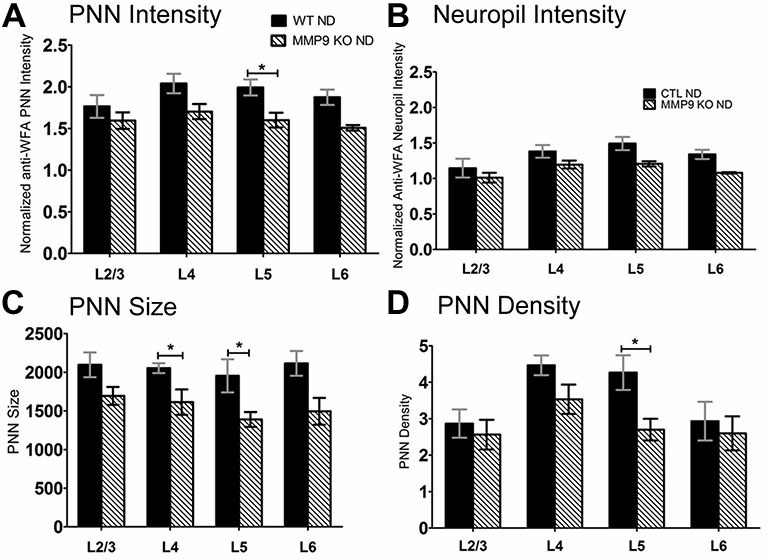
**CSPGs composition comparisons between WT and matrix metalloproteinase 9 (MMP9) knock-out (KO) mice. (A–D)** Quantitative analysis of PNN intensity, Neuropil intensity, PNN size, and PNN density between WT and MMP9 KO ND mice showing few differences between the ND conditions. Significant decreases were noted in MMP9 KO mice in L5 of PNN intensity, PNN size and PNN density suggesting a laminar effect of MMP9 depletion. Statistics = Two-way ANOVA with Bonferroni multiple comparisons *post hoc* analysis. All values reported are the mean ± SEM. *p < 0.05.

**Figure 4:**
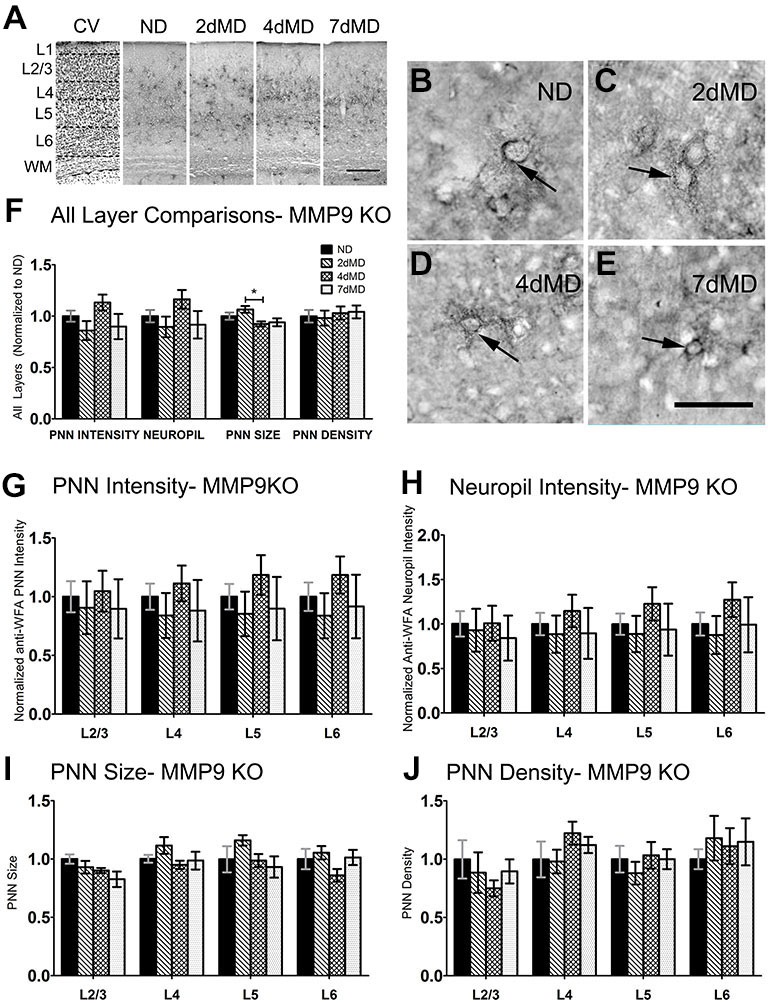
**CSPG degradation is absent in MMP9 KO mice. (A)** Representative examples of low-mag (4X) images showing laminar demarcation in CV alongside CSPG (anti-WFA) immuno-reactive tissue in MMP9 KO mice. **(B–E)** Anti-WFA immunoreactivity labels CSPG deposits in PNNs (arrows) in all experimental conditions in MMP9 KO mice. **(F)** Quantitative analysis of several labeling parameters in all layers across experimental conditions. Values were normalized to ND controls for presentation purposes only. Little degradation is apparent following MD. **(G–J)** Quantitative analysis of individual labeling parameters across layer. No significant degradation of CSPG immunoreactivity was evident suggesting that the lack of MMP9 attenuated the effects of MD on extracellular matrix (ECM) degradation. Scale bar **(A)** = 250 μm; **(B–E)** = 50 μm. Statistics = Two-Way ANOVA with Bonferroni multiple comparison *post hoc* analysis. All values reported are the mean ± SEM. *p < 0.05.

ECM degradation is regulated by proteases (such as MMPs) secreted by both neurons and glia (Webster and Crowe, 2006; Dziembowska and Wlodarczyk, 2012; Konnecke and Bechmann, 2013), and this process is thought to promote synaptic plasticity (Mataga et al., 2002, 2004; Pizzorusso et al., 2002; Oray et al., 2004). MMP9 in particular is highly expressed in the brain, and is rapidly upregulated during plasticity (Szklarczyk et al., 2002). In the somatosensory cortex, MMP9 activity is upregulated following whisker deprivation and affects experience-dependent barrel remodeling (Kaliszewska et al., 2012), suggesting that a similar mechanism could be used in the visual cortex. We first examined whether CSPG composition in the primary visual cortex was altered by loss of MMP9 (Figure 3). Analysis of PNN intensity, neuropil intensity, PNN size and PNN density (Figures 3A–D, two-way ANOVA with Bonferroni multiple comparisons *post hoc* analysis) showed a similar deposition and distribution of CSPGs in the visual cortex of WT and MMP9 KO mice. While lower levels of PNN and neuropil staining, PNN size and density were observed in MMP9 KO mice (two-way ANOVA, effect of genotype *p* < 0.05), few of these changes reached statistical significance and were generally restricted to layer 5 (Bonferroni *post hoc* analysis; *p* < 0.05). The magnitude of the changes was also small compared to the degradation observed following MD (Figure 2), suggesting that developmental loss of MMP9 has a limited effect on CSPG regulation. To determine if MD-induced ECM degradation was mediated by MMP9, we monocularly deprived MMP9 KO mice for 2, 4, and 7 days and assayed CSPG degradation using anti-WFA immunoreactivity. CSPG deposition in MMP9 KO mice was observed in dense accumulations around cell bodies, as well as dense labeling in the neuropil in all deprivation conditions (Figures 4A–E) as in WT animals. In contrast to the ECM degradation we observed in control animals following MD (Figure 2), CSPG composition remained intact in MMP9 KO mice (Figures 4G–J), suggesting that MMP9 contributes to ECM remodeling during ODP.

**Figure 5:**
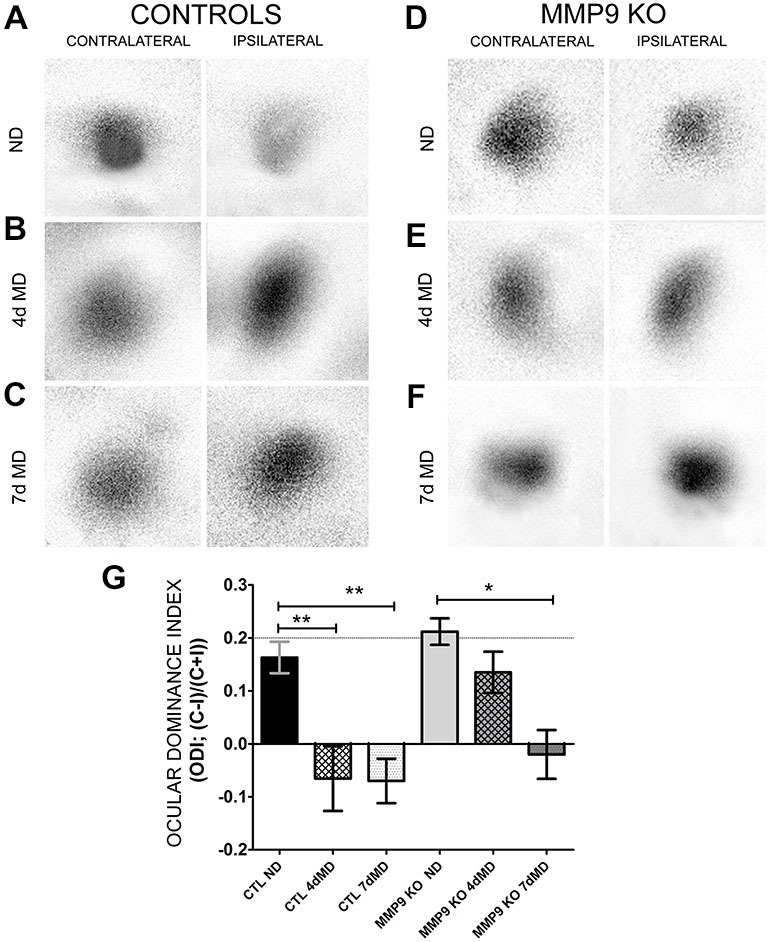
**Ocular dominance plasticity (ODP) is attenuated in MMP9 KO mice. (A–F)** Representative examples of visually-evoked amplitude maps measured using intrinsic signal optical imaging (iOS) in control (C57Bl/6) and MMP9 KO mice. **(G)** Quantitative analysis of ODP comparing changes in the ocular dominance index (ODI). Values above 0 denote an ODI with a contralateral bias, while values below value suggest an ODI with an ipsilateral bias. CTL ND (black bar) have a characteristic and expected contralateral bias that significantly shifts to an ipsilateral bias after both 4dMD (CTL 4dMD, white hatched bar) and 7dMD (CTL 7dMD, white dotted bars). MMP9 KO ND (gray bar) displayed a contralateral bias, similar to CTL ND. Following 4dMD (gray hatched bar), MMP9 KO mice failed to demonstrate an ODP shift. ODP was apparent in MMP9 KO 7dMD (gray dotted bar). Statistics = Two-Way ANOVA with bonferroni multiple comparisons. All values reported are the mean ± SEM. *p < 0.05, **p < 0.001.

Next we wanted to determine whether MMP9-mediated ECM degradation contributes to functional OD shifts. Therefore we performed intrinsic signal optical imaging (iOS) on control (C57Bl/6; CTL) and MMP9 KO mice following 4 days of monocular deprivation (4dMD) and quantified binocularity by calculating an ODI. An ODI above 0 represent a contralateral bias, while values below 0 represent an ipsilateral bias (Cang et al., 2005). CTL mice showed the expected contralateral bias in the absence of MD (Figures 5A,G, black bar, 0.163 ± 0.03). Following MD, responsiveness shifted from a contralateral to ipsilateral bias (Figures 5B,G, white hatched bar (−0.06 ± 0.06), white dotted bar (−0.07 ± 0.04). Non-deprived MMP9 KO mice also showed a strong contralateral response, with an ODI comparable to CTL ND mice (Figures 5D,G, gray bar, 0.212 ± 0.02). After 4 days of MD, however, ODIs failed to shift towards the ipsilateral eye (Figures 5E,G, gray hatched bar, 0.135 ± 0.04). To determine whether plasticity was reduced or slowed by the absence of MMP9, we repeated the experiment after 7 days of MD. By 7dMD, MMP9 KO mice displayed a significant ipsilateral shift similar to that observed in CTL 7dMD mice (Figures 5C,F,G, gray dotted bar, −0.02 ± 0.04, *p* < 0.05, two-way ANOVA, Bonferroni multiple comparisons) suggesting that MMP9 deficiency delays the induction or manifestation of ODP.

The effects of MMP9 KO on plasticity may be explained by the lack of ECM remodeling following deprivation or by other changes that may occur in the brain due to developmental loss of MMP9 that can leave the cortex in a less plastic state. Our finding that CSPG deposition is altered to a small extent in MMP9 KO mice, suggests that developmental changes in the ECM are unlikely to limit plasticity. However, MMPs can have profound effects on the development of neural circuits (Aerts et al., 2015), as well as the function of other cell types in the CNS (Hansen et al., 2013). Therefore we decided to further characterize the MMP9 KO mice to determine what other changes could contribute to the blunted experience-dependent plasticity we observed. We decided to focus specifically on dendritic spine development and microglial phenotypes, both of which have been shown to be critical for visually-driven plasticity (Oray et al., 2004; Tremblay et al., 2010a).

**Figure 6:**
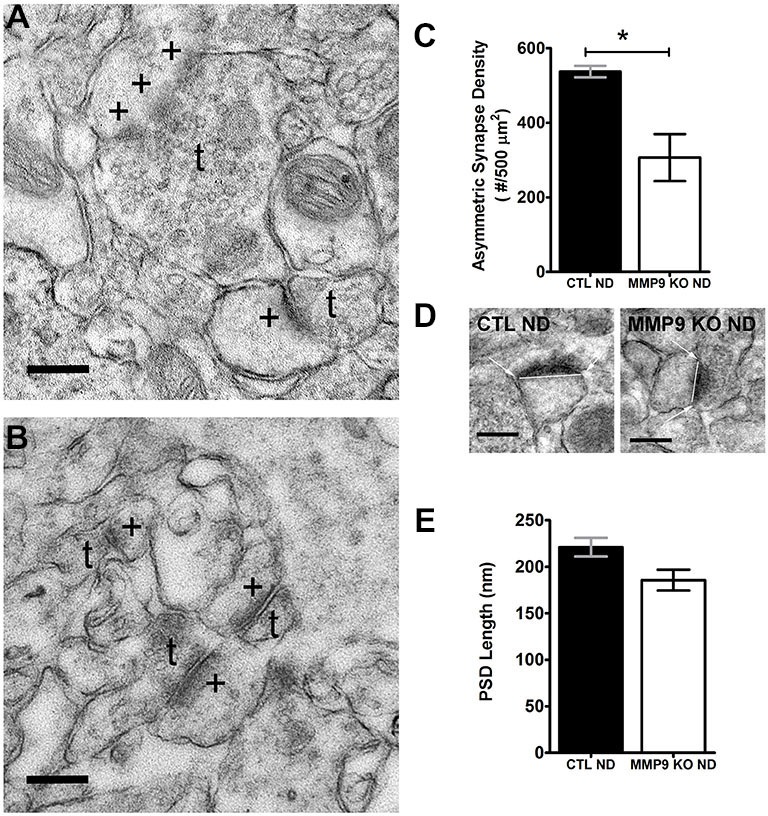
**Synapse Density but not postsynaptic density (PSD) size is affected in MMP9 KO mice.** Representative examples of images obtained from CTL ND **(A)** and MMP9 KO ND **(B)** visual cortex following pre-embedding electron microscopy ultra-structural preparation for synaptic profiling. Images show the appearance of asymmetric (excitatory; glutamatergic) synapses (denoted by *+*) abutting presynaptic terminals containing neurotransmitter vesicles (denoted by *t*). **(C)** Quantitative analysis of the total number of asymmetrical synapses in CTL ND (black bar) vs. MMP9 KO ND (white bar) showing a significant decrease in MMP9 KO synapses. **(D)** Examples of PSD length measurements performed using Image J software in CTL ND (first panel) and MMP9 KO ND (second panel). **(E)** Quantitative analysis of PSD lengths in CTL ND (black bar) and MMP9 KO ND (white bar). No significant difference was observed. Scale bars = 200 nm **(A,B)**; 250 nm **(D)**. Statistics = Students *t*-test. All values reported are the mean ± SEM. *p < 0.05.

MMP9 and the ECM can regulate the structure of dendritic spines, the postsynaptic sites of excitatory synapses, (Szklarczyk et al., 2002; Ethell and Ethell, 2007), and dynamic changes in dendritic spine structure accompany ODP (Oray et al., 2004). To ascertain whether changes in ECM composition in MMP9 KO mice may differentially regulate cortical excitatory synapses and thus affect plasticity, we used electron microscopy to quantify asymmetric synapse density (Figures 6A–C) and PSD size (Figure 6D) in layer 2 of visual cortex. We found that MMP9 KO mice had significantly fewer asymmetric synapses in the same cortical area when compared to CTL mice (Figure 6C). This change in synaptic density was not accompanied by changes in PSD size (Figure 6E). These data suggest that deficits in excitatory synapse development may contribute to the plasticity phenotype observed in MMP9 KO mice, and that synaptic density rather than synaptic strength may be affected.

**Figure 7:**
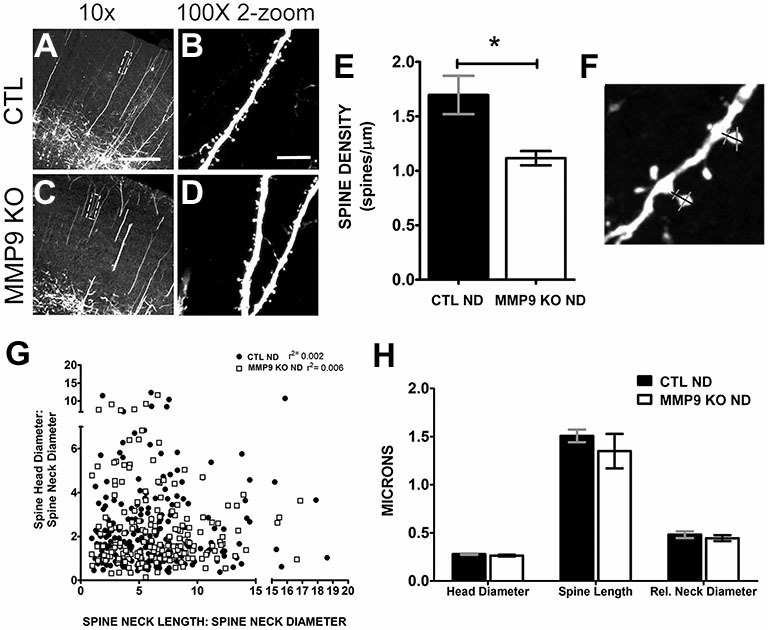
**Dendritic spine density and morphology are differentially affected in MMP9 KO mice.** Representative confocal images of CTL **(A,B)** and MMP9 KO **(C,D)** cortical dendrites of layer 5 pyramidal neurons. **(E)** Quantitative analysis of spine density showed significantly more spines in CTL ND (black bar, 1.697 ± 0.176) compared to MMP9 KO mice (white bar; 1.116 ± 0.065; Student’s *t*-test with Mann Whitney test, *p < 0.05). **(F)** Example of spine measurements. Spine neck length (black bar) and spine head and neck width (gray bars) measurements were made using Image J software. **(G)** Correlation analysis depicting no differences between head and neck comparisons across genotypes (Linear regression analysis; CTL ND *r*^2^ = 0.002, MMP9 ND *r*^2^ = 0.006). **(H)** Head diameter, spine length, and relative neck diameter were comparable between the two genotypes (Two-way ANOVA with bonferroni multiple comparisons). Scale bar = 200 μm. All values reported are the mean ± SEM.

To further explore the differences in synaptic behavior caused by loss of MMP9, we crossed MMP9 KO mice with GFP-M transgenic mice (Feng et al., 2000) in which GFP is expressed in L5 pyramidal neurons (Figures 7A–D). Because GFP expression is low in visual cortex during adolescence we assayed dendritic spine morphology in S1, a brain area in which dendritic spine development mirrors that in primary visual cortex (Elston and Fujita, 2014). Confocal microscopy revealed GFP-labeled dendritic branches in both GFP-M (CTL ND, Figure 7A) and MMP9 KO-GFP (MMP9 KO, Figure 7C) adolescent (P28) S1, with clearly discernable dendritic spines on the apical dendritic branches (in L2/3) in both genotypes (Figures 7B,D). As the formation and elimination of synapses is believed to be one of the mechanisms underlying adaptive remodeling of neural circuits (Trachtenberg et al., 2002; Majewska et al., 2006) and previous reports implicate a role for MMP9 on dendritic spine development (Szklarczyk et al., 2002; Tian et al., 2007; Stawarski et al., 2014), we wanted to know how the density, structure and dynamics of dendritic spines were affected by MMP9 loss. We first wanted to determine whether dendritic spines in S1 were regulated similarly to those in V1. Therefore, we first compared changes in synaptic density between GFP-M and MMP9 KO-GFP mice in S1 to see if initial development of networks was compromised. We found a significant reduction in spine density in MMP9 KO mice (Figure 7E, *p* < 0.05), consistent with the decrease in asymmetric synapse density observed in V1 (Figure 6C). This suggests that MMP9 may have similar roles in synaptic development across sensory cortical areas. Since spine density changes are often associated with alterations in spine morphology (Wallace and Bear, 2004), we measured the dimensions of each spine, including spine head and relative neck diameter (Figure 7F, gray bars) and dendritic spine length (Figure 7F, black bar measuring from dendritic shaft to tip of protrusion). Changes in spine shape can predict spine developmental profiles; where thinner spine heads and longer necks suggest an immature phenotype while a wider head and short neck might suggest maturity (reviewed in Sala and Segal, 2014). Interestingly, while we noted a significant reduction in dendritic spine density in MMP9 KO mice, we found no differences in the distribution of spine “types”, as spine head and neck comparisons did not differ across genotypes and these comparisons correspond to relative distributions of mushroom, thin and stubby spines (Figure 7G). Similarly, spine morphology was unchanged when compared to GFP control mice (Figure 7H) suggesting no clear changes in the morphological classes of spines present in GFP CTL and MMP9 KO GFP mice, supporting the data obtained in visual cortex showing no difference in PSD size between the two genotypes.

**Figure 8:**
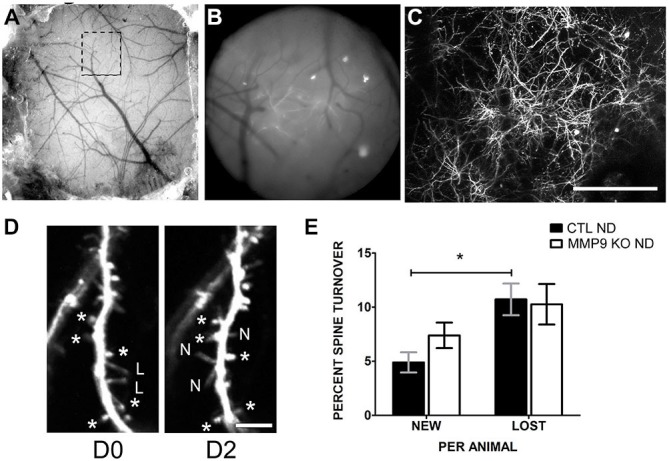
**Dendritic spine turnover is altered in MMP9 KO mice. (A)** Example of thin skull preparation viewed through dissecting scope showing clear demarcation of brain vasculature. **(B)** Magnified epifluorescent image of the region of interest (ROI, hatched box in **(A)**) visualized with a 20 × 0.95 NA objective lens. Vasculature landmarks aided in relocating ROI in chronic imaging. **(C)** Two-photon image of the same region (as **A** and **B**) taken at 1× digital zoom which served as a reference map of magnified vessels and dendritic branches. **(D)** 8x digital zoom of dendritic branches and spines on D0 (first panel) and identical branches at D2 (second panel). Asterisks (d*) denote reference spines between images. L = lost spines, N = new spines. **(E)** Quantitative analysis of percent dendritic spine turnover in CTL ND (black bars) and MMP9 KO ND (white bars) mice. CTL ND mice showed significantly more lost spines than gained spines after 2 days (Two-Way ANOVA, *p < 0.05). Spine loss and gain were not significantly different in MMP9 KO ND mice. Scale bar = 200 μm **(A–C)**, 5 μm **(D)**. All values reported are the mean ± SEM.

Alterations in spine density may be the result of aberrant spine formation or elimination. To determine if spine dynamics are affected by MMP9 deficiency, we investigated dendritic spine turnover in GFP-M CTL mice and MMP9 KO GFP *in vivo* using chronic two-photon (2P) imaging. The skull over S1 was thinned to reveal the cortical vasculature (Figures 8A–C) which was used as a reference for chronic imaging. Dendritic spines from the apical tufts of L5 pyramidal neurons were imaged on D0 (first day imaging, P28) and the same spines were reimaged 2 days later (D2) (Figure 8D). Under basal conditions (CTL ND), CTL GFP-M mice displayed a significantly greater rate of spine loss compared to spine gain (Figure 8E, black bars, *p* < 0.05), as has previously been described during adolescence (Zuo et al., 2005). This effect was not seen in mice lacking MMP9, where loss and gain rates were well matched (Figure 8E, white bars). These results suggest that spine dynamics are altered in MMP9 KO mice.

**Figure 9:**
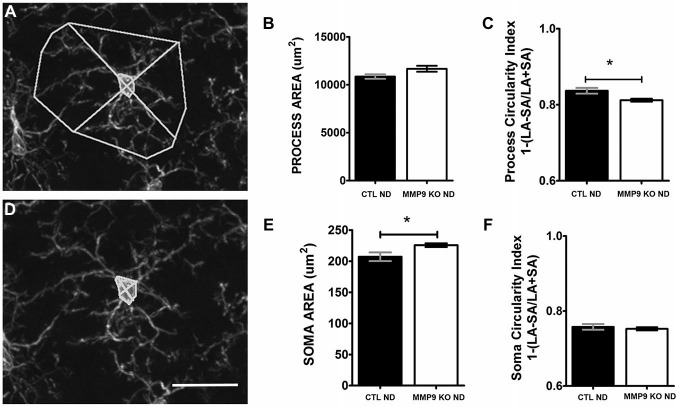
**MMP9 KO mice show changes in microglial morphology. (A)** The area occupied by microglial processes was measured using Image J software. **(B)** Quantitative analysis of microglial process area was not significantly different between CTL ND (black bars, 0.837 ± 0.008) and MMP9 KO ND (white bars, 0.812 ± 0.004; unpaired student’s *t*-test). **(C)** Process circularity index [1−(LA−SA)/(LA+SA)] was significantly decreased in MMP9 KO ND mice (unpaired student’s *t*-test, *p < 0.05) **(D)** The area of microglial soma was measured using Image J software. **(E)** Quantitative analysis of the soma area showed significantly larger microglial soma in MMP9 KO ND (unpaired student’s *t*-test, *p < 0.05) with no significant changes in overall soma circularity **(F)** Scale bar = 20 μm. All values reported are the mean ± SEM.

Microglia secrete proteases (Webster and Crowe, 2006; Konnecke and Bechmann, 2013), including MMP9, that contribute to localized proteolytic activity during physiological and pathological events. Furthermore, microglial interactions with synapses have recently been shown to promote healthy brain homeostasis, including the regulation of cell death, synapse elimination, neurogenesis, and neuronal surveillance (Paolicelli et al., 2011; Tremblay et al., 2011; Wake et al., 2013). Thus MMP9 depletion may alter microglia morphology and function and influence dendritic spine pruning (Szklarczyk et al., 2002). To determine the effects of MMP9 deficiency on microglia, we first investigated changes in microglial morphology as these can indicate changes in microglial function. Whereas pathological microglial activation is characterized by the thickening, polarization and retraction of branches, as well as an increase in soma size, a resting/ quiescent state is characterized by a ramified, circular arbor and small soma (Hanisch and Kettenmann, 2007; Kettenmann et al., 2011). CTL and MMP9 KO tissue from the binocular visual cortex was processed for the anti-microglial marker, Iba-1, visualized with immunofluorescence and analyzed using confocal microscopy. Confocal *z*-stack images allowed us to fully assess the extent of microglial process arbor (Figure 9A) and soma (Figure 9D). While we noted no significant difference in the area occupied by the microglial process arbor in CTL and MMP9 KO microglia (Figure 9B), we found a small but significant decrease in the process circularity index (Figure 9C, *p* < 0.05, Student’s *t*-test) signifying arbor elongation in MMP9 KO microglia. MMP9 KO microglia also had a small but significant increase in soma size (Figure 9E, *p* < 0.05, Student’s *t*-test) but not soma circularity (Figure 9F). These results suggest that MMP9 deficiency has a limited effect on microglial morphology but the morphological changes observed suggest a more activated state.

**Figure 10:**
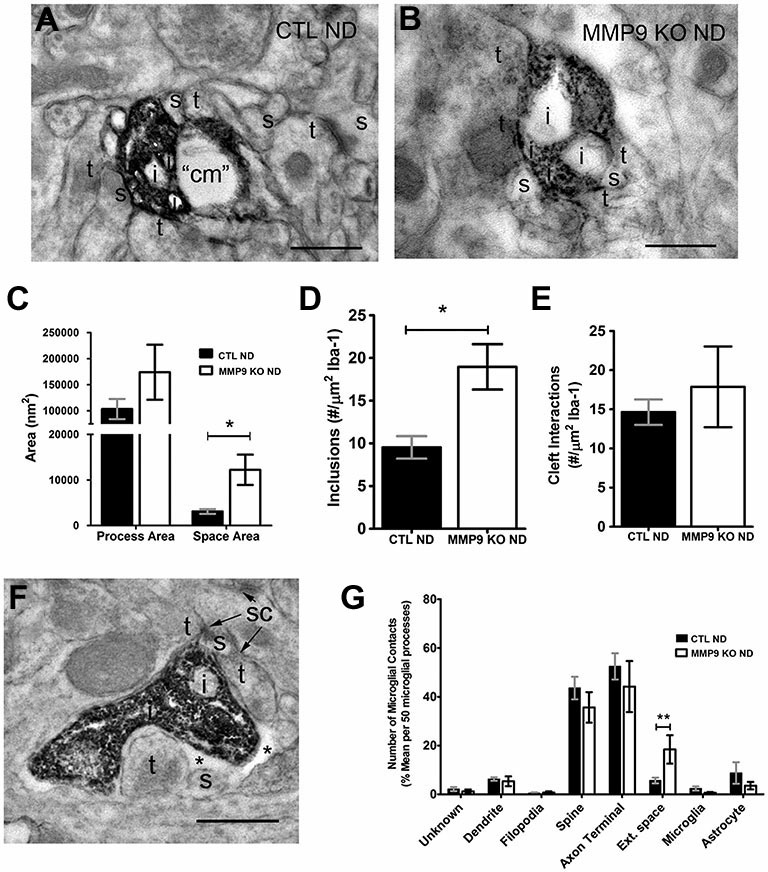
**Ultrastructural analysis of microglia suggest mild activation in the absence of MMP9. (A,B)** Iba-1 immunoperoxidase EM in non-deprived control mice (CTL ND) and non-deprived MMP9 KO mice (MMP9 KO ND). **(C)** Quantitative analysis of microglial process area showed no significant differences, while surrounding extracellular space was significantly increased in MMP9 KO ND compared to CTL ND (One-Way ANOVA, *p < 0.05). **(D)** Average number of inclusions per area of Iba-1 labeling in 50 microglia per animals demonstrating a significant increase in inclusions in MMP9 KO ND mice (unpaired student’s *t*-test, *p < 0.05). **(E)** Average number of synaptic contacts per area of Iba-1 labeling in 50 microglia per animals showing no significant differences between CTL ND and MMP9 KO ND mice. **(F)** Iba-1 immunoperoxidase EM showing close apposition of a microglial process to excitatory synaptic sites (sc). **(G)** Quantification of neighboring contacts to Iba-1 immunoreactive microglial processes. We found no significant differences between microglial contacts which showed similar interactions with spines and terminals in both CTL and MMP9 KO mice (One-Way ANOVA, **p < 0.001). All values reported are the mean ± SEM. t, axon terminal; s, dendritic spine; i, inclusion; cm, cellular membrane; sc, synaptic contact; *, extracellular space. Scale bar = 500 nm.

To further investigate the potential activation of microglia, we preformed pre-embedding immuno-peroxidase electron microscopy on samples taken from the primary visual cortex at P28 in both control C57BL/6 and experimental MMP9 KO mice. Sections were processed for Iba-1 immunoreactivity, resulting in clear demarcation of microglial processes, internal content and neighboring elements in both control (CTL ND, Figure 10A) and MMP9 KO mice (MMP9 KO ND, Figure 10B). We observed no difference in microglial process size in MMP9 KO mice, but found significantly larger pockets of extracellular space surrounding microglia (Figure 10C). Furthermore, we found significantly more inclusions (intracellular vacuoles often containing engulfed cellular content) in the MMP9 KO (Figure 10D). To determine whether this increase in inclusions was due to changes in microglial behavior towards synapses (Paolicelli et al., 2011), we quantified microglial contacts with stereotypic excitatory synapses. We measured the degree of microglial contact with excitatory pre- and postsynaptic terminals (characterized by synaptic vesicle accumulation in the presynaptic terminal abutting a postsynaptic terminal containing a visible PSD) often surrounding the synaptic cleft. We found no differences in microglial contacts with the synaptic cleft (Figure 10E) suggesting that increased phagocytosis in MMP9 KO microglia may not be specific to synapses. We also found a similar profile of structures that contacted microglial processes in CTL or MMP9 KO microglia (aside from a significant increase in the number of pockets of extracellular space surrounding MMP9 KO microglia), further suggesting a lack of microglial targeting to specific elements in MMP9 KO mice as compared to CTL (Figures 10F,G).

## Discussion

In this study, we analyzed the role of MMP9, one of the primary MMPs in the brain, in experience-dependent plasticity. We show that several components of the ECM are differentially regulated following MD in the rodent primary visual cortex, and that the degradation of CSPGs after MD is not present in MMP9 KO mice. ODP was also attenuated in MMP9 KO mice following MD showing that MMP9 is an important player in experience-dependent plasticity. Inspection of excitatory synapses and dendritic spines revealed significantly lower asymmetric synapse density in layer 2, and lower spine density on the apical tufts of layer 5 pyramidal neurons in MMP9 KO mice with no change in spine morphology. MMP9 KO mice also displayed defects in dendritic spine turnover. The effect of MMP9 deficiency on microglial morphology was small although increases in microglial inclusions in MMP9 KO mice suggest mild redirection of microglial function towards an activated phenotype. Collectively, our findings suggest an instrumental role for MMP9 in activity-dependent plasticity in the rodent sensory cortex.

### The Extracellular Matrix Plays a Fundamental Role in the Regulation of Activity-Dependent Plasticity

Synaptic plasticity in the brain involves a complex series of interactions that enable the reorganization of connections as a response to sensory experience. In the rodent visual cortex, deprivation (via lid suture) triggers circuit reorganization that enables strengthening of intact inputs and weakening of deprived circuits (Gordon and Stryker, 1996; Frenkel and Bear, 2004). Plasticity occurs at the level of dendritic spines, the small protrusions originating from dendritic shafts, which contain synaptic machinery and are the primary sites of interneuronal communication (Holtmaat and Svoboda, 2009). Plasticity can be implemented through changes in spine number, spine shape, and signaling strength (Sala and Segal, 2014). Recent studies have shown that spine structure can be regulated by the ECM (reviewed in Ethell and Ethell, 2007; Levy et al., 2014; Stawarski et al., 2014) and that MMPs play a fundamental role in regulating ECM degradation and signaling to enable plasticity.

The ECM in the neuropil and in the PNN, a specialized extracellular structure formed around certain neurons, can form a barrier around maturing neurons, and limit circuit reorganization by (1) preventing interaction with neurons and advancing axons; (2) providing a scaffold for inhibitors; and (3) limiting receptor mobility at the synapse (Wang and Fawcett, 2012). Degradation of the ECM can reactivate plasticity in adults (Pizzorusso et al., 2002) suggesting that ECM composition is an important factor in activity-dependent plasticity. HA comprises a fundamental base of the ECM lattice in the neuropil and in the PNN and the tightness of this lattice can be altered by HA content and the proteins that bind HA (Yamaguchi, 2000). HSPGs are present in the neuropil and in PNNs and are important for binding various molecules that could affect synaptic plasticity. CSPGs (lecticans) versican, brevican, neurocan, CAT-301 antigen (aggrecan), phosphacan and link proteins (Hockfield and McKay, 1983; Celio and Blümcke, 1994; Celio et al., 1998) are strongly present in PNNs where they are involved in multiple processes, including regulation of neuronal plasticity (Pizzorusso et al., 2002; Spolidoro et al., 2012), and neuroprotection (Brückner et al., 1999; Morawski et al., 2004). The composition of the lecticans within the PNN is a fundamental determinant of ECM structure and functionality (Yamaguchi, 2000). Therefore, understanding how these different ECM components are regulated within PNNs and in the neuropil surrounding synapses that are undergoing remodeling after MD is important to untangling the function of the ECM during ODP.

In the visual cortex, HA and HSPGs showed primarily neuropil staining with occasional staining around cell bodies, while CSPGs were present in the neuropil and densely within PNNs. In the neuropil, we observed a trend towards degradation of HA throughout the cortical layers and the time scale of deprivation with no changes in HSPG content (Figure 1), but significant degradation of neuropil CSPGs in all layers beginning at 2dMD (Figure 2). These findings suggest that the ECM is remodeled relatively early in ODP when responses from the deprived eye are weakened (Frenkel and Bear, 2004), and when synaptic rearrangement and local upregulation of proteases occurs (Mataga et al., 2002, 2004; Oray et al., 2004). Degradation of HA, in the absence of HSPG remodeling, may be sufficient to affect the overall “tightness” of the ECM (Yamaguchi, 2000) where the amount of HA, the structural makeup of its domains, and affinity for binding other lecticans all contribute to the fluidity of the structure. This in turn may lead to delayed remodeling of associated CSPGs which could impact the strengthening of non-deprived eye responses. Interestingly, we also noted a significant decrease in CSPG immunoreactivity within PNNs (Figure 2), suggesting that neuropil and PNN ECM content is regulated in a similar manner. While PNN size was relatively unaffected by MD, PNN density increased in layer 2/3 possibly to compensate the degradation of CSPGs throughout cortex, Interestingly, MMP9 loss only mildly affected the distribution of CSPGs in the absence of deprivation suggesting that MMP9 does not play a large role in the development of ECM structure or that its loss is compensated in these mice through other mechanisms.

MD-induced degradation of CSPGs was abolished in MMP9 KO mice, suggesting that CSPG content is regulated by MMP9. It is likely that this regulation is coupled with the effects of tPA activity that has been shown to be upregulated following MD (Muller and Griesinger, 1998; Mataga et al., 2002, 2004), especially given that exogenous application of tPA can mimic the structural effects of ODP (Oray et al., 2004). Furthermore, the conversion of the pro-MMP9 zymogen (inactive) to active MMP9 lies downstream of tPA/plasmin activation (Ramos-DeSimone et al., 1999) suggesting that MD induces a pathway in which pro-MMP9 is cleaved and active MMP9 activates the tPA/plasmin axis to implement changes in the ECM.

### Regulation of Plasticity by Matrix Metalloproteinases 9 (MMP9)

MMPs belong to a family of zinc-dependent endopeptidases that are crucial effectors in the development and remodeling of various tissues (Sternlicht and Werb, 2001). MMP9 is the best-characterized MMP family member involved in activity-dependent structural and functional changes at CNS synapses. Increased neuronal activity enhances MMP9 expression in an NMDA-dependent manner (Sternlicht and Werb, 2001; Szklarczyk et al., 2002; Nagy et al., 2006) and MMP9 has been shown to regulate late phase LTP *in vivo* (Nagy et al., 2006), learning and memory (Bozdagi et al., 2007), experience-dependent plasticity (Kaliszewska et al., 2012), and dendritic spine morphology (Tian et al., 2007; Michaluk et al., 2011). Indeed, the synaptic localization of MMP9 implies a fundamental role in neuronal plasticity.

In the visual cortex, ODP was attenuated in MMP9 KO mice following 4dMD but not 7dMD (Figure 5) suggesting an effect of MMP9 during specific phases of ODP. Following closure of one eye, ODP occurs in a biphasic manner, in which first there is a decrease in deprived eye responses, followed by increased potentiation in the ipsilateral non-deprived eye (Frenkel and Bear, 2004). Our results suggest that MMP9 affects initial phases of ODP, consistent with results seen in tPA KO mice (Mataga et al., 2002), and in ECM changes described in this study which occur at 2dMD. Alternatively, MMP9 KO may delay the effects of MD, possibly due to an inhibitory ECM milieu, and require longer deprivations to elicit plasticity. In contrast, previous reports suggest that MMP9 inhibition (infusion of the global MMP inhibitor, GM6001) only affects the potentiation of the open-eye responses due to the attenuating effects of MMP9 on ODP following 7dMD only (Spolidoro et al., 2012). An important difference between the two studies is the approach to abrogating MMP activity. Our model is less invasive and allows for complete loss of MMP9 activity without off-target effects, whereas GM6001 is a non-specific MMP inhibitor. Our genetic model, however, potentially allows for developmental compensation for MMP9 loss. In addition, the use of a mouse model (this study) vs. rat model (Spolidoro et al., 2012) may introduce differences in plasticity mechanisms (Kadish and Van Groen, 2003). While more work will be needed to reconcile these different results, collectively, it is clear that MMP9 is necessary for specific forms of experience dependent plasticity that may vary depending on the experimental design.

While our data suggests that MMP9 plays important role in the remodeling of the ECM and that this remodeling may be a critical step in ODP, MMP9 loss during development likely alters other pathways that could impact plasticity. To explore this possibility we further characterized the MMP9 KO cortex for other changes which could lead to an altered potential for ODP. MMP9 is important in late-phase LTP (Nagy et al., 2006; Wang et al., 2008); a process often accompanied by changes in dendritic spine density and morphology (Yuste and Bonhoeffer, 2001). In our study, MMP9 KO mice also exhibited reduced excitatory synapse density in V1 and reduced spine densities on the apical dendrites of S1 layer 5 neurons, without changes in PSD size or spine morphology, respectively (Figure 7). MMP9-mediated cleavage of ECM/CAM (cell adhesion molecules) results in products that can act on integrin receptors to transduce the signals provoking actin cytoskeleton modification (reviewed in Wlodarczyk et al., 2011, but also Nyman-Huttunen et al., 2006), suggesting that a reduction in spine density in MMP9 KO mice maybe due to a defect in signaling that regulates spine formation and elimination during early periods of development. MMP9 KO mice also do not show increased elimination of dendritic spines as compared to spine formation (Figure 8E), which is a hallmark of normal adolescent cortex. This may suggest that a compensatory mechanism is activated to counteract the low density of spines by normalizing spine formation and elimination.

Despite decreased overall excitatory synapse density in MMP9 KO mice, PSD size and spine morphology (Figures 6E, 7G–H) was unaffected indicating that while the initial mechanisms of synapse formation may be disrupted, synapses that are formed continue to mature normally. This was unexpected to us as MMP9 has been reported to modulate dendritic spine morphology (Tian et al., 2007; Wang et al., 2008; Bilousova et al., 2009; Michaluk et al., 2011; Dziembowska et al., 2013). In transgenic rats overexpressing an autoactivating mutant of MMP9, dendritic spines appeared thinner and longer (Michaluk et al., 2011). Similarly, in the fragile X mouse model, there was an increase in the ratio of filopodia to mature spines; reversed by the MMP9 inhibitor, minocycline (Bilousova et al., 2009). MMP9 overexpressing mice also showed a preponderance of immature spine phenotypes (Gkogkas et al., 2014), while MMP9 KO mice have previously been described to have longer and larger spines (Sidhu et al., 2014), although this effect was observed only earlier in development. The regulation of ICAM-5, a telencephalin-associated intracellular adhesion molecule, by MMP9 (and other MMPs) provides a compelling link between MMP9 and spine morphology in which cleavage of ICAM-5 by MMPs is able to regulate spine maturation (Tian et al., 2007; Conant et al., 2010). In these studies, MMP9 appeared to have a pleiotropic effect, resulting in both elongation of filopodia as well as maturation of spine heads. Interestingly, the soluble (cleaved) portion of ICAM-5 (sICAM-5) can also bind to β1 integrin receptors on nascent spines, resulting in the phosphorylation of cofilin and triggering spine growth (Conant et al., 2011; Ning et al., 2013). Indeed, MMP9-dependent synaptic effects through β1 integrin receptors signaling suggest that MMP9 cleaves ECM proteins with exposed integrin activating epitopes (cryptic RGD motifs), resulting in the surface diffusion of NMDA receptors (Michaluk et al., 2009). Taken together with the results of our study, these data show that MMP9 has multiple effectors that contribute to regulating dendritic spine morphology in a complex manner, however effects on synapse density and plasticity can be seen in the absence of changes in spine morphology. Additionally, our results suggest that altered development of visual cortical circuitry in the absence of MMP9, along with the inability to remodel the ECM in response to deprivation, may be responsible for blunted ODP in MMP9 KO mice.

### Matrix Metalloproteinases and Microglia

Microglia are the resident immune cells of the brain and are instrumental in pathological responses. Classically thought to play a pivotal role during onset, maintenance, relapse and progression of inflammatory conditions (Kettenmann et al., 2011; Konnecke and Bechmann, 2013), microglia have recently been shown to contribute to mechanisms that regulate neurodevelopmental processes, including synaptic interactions and remodeling (Wake et al., 2009, 2013; Paolicelli et al., 2011; Tremblay and Majewska, 2011; Tremblay, 2011). Microglia secrete MMP9 (Webster and Crowe, 2006; Konnecke and Bechmann, 2013) as well as other proteases and protease inhibitors, although the role of MMP9 can vary depending on the condition studied. In injury models, inflammation triggers increases in MMP9 and the proinflammatory cytokine, TNF-α, at injury sites (Hansen et al., 2013). Indeed, the upregulation of MMPs in the CNS have several potentially detrimental roles, including the promotion of neuroinflammation, disruption of the blood brain barrier, demyelination, and damage to axons and neurons (reviewed in Konnecke and Bechmann, 2013). Secretion of MMP9 from microglia can be beneficial as well, contributing to ECM degradation around synaptic sites and promoting synapse reorganization and functional recovery following traumatic brain injury (Chan et al., 2014). Although MMP9 inhibition has been reported to attenuate microglia activation (Hansen et al., 2013), here we show that in MMP9 KO mice, microglial morphology is largely unaltered with some evidence of changes which suggest a change in microglial phenotype towards a more activated state (decrease in process circularity (Figure 9C) and an increased number of inclusions (Figure 10D)). This may be a direct result of MMP9 loss or the result of compensatory upregulation of other proteases (Sekine-Aizawa et al., 2001; Esparza et al., 2004; Greenlee et al., 2007). Thus changes in microglia are subtle in MMP9 KO mice, although these changes might also contribute to altered plasticity in these mice.

## Conclusion

MMPs belong to a large family of endopeptidases shown to be instrumental regulators in both physiological and pathological events throughout the PNS and CNS (Sternlicht and Werb, 2001). Their biochemical complexity and the large variety of substrates accounts for the richness of their functions (Ethell and Ethell, 2007). In this study we show that MMP9 may contribute to early stages of ODP. MMP9 affects many neuronal and glial processes and its effects on ODP may result from a combination of ECM degradation, remodeling of excitatory synapses, and changes in microglia function. Given evidence that proteases act in a complimentary and often redundant manner (Sekine-Aizawa et al., 2001), future studies should investigate the interaction between MMP9 and other closely associated molecules such as tPA and MMP2 during plasticity.

## Conflict of Interest Statement

The authors declare that the research was conducted in the absence of any commercial or financial relationships that could be construed as a potential conflict of interest.

## Footnotes

^1^Freeware: http://rsb.info.nih.gov/ij/

^2^http://graphpad.com/quickcalcs/Grubbs1.cfm
